# Beyond therapeutic potential: a systematic investigation of ketamine misuse in patients with depressive disorders

**DOI:** 10.1007/s44192-024-00077-2

**Published:** 2024-07-01

**Authors:** Keshav Juneja, Sabah Afroze, Zeel Goti, Sweta Sahu, Shivani Asawa, Hamsa Priya Bhuchakra, Balaganesh Natarajan

**Affiliations:** 1 B.J. Medical College, Ahmedabad, India; 2 Shadan Hospital and Institute of Medical Sciences, Hyderabad, India; 3 Government Medical College, Surat, India; 4 JJM Medical College, Davanagere, India; 5 Apollo Institute of Medical Sciences & Research, Hyderabad, India; 6 St. George’s University, True Blue, Grenada

**Keywords:** Ketamine, Major depressive disorder, Abuse, Misuse, Rapid-acting antidepressants, Ketamine treatment

## Abstract

Ketamine, a pharmacological agent that acts as an antagonist of the N-methyl-D-aspartate (NMDA) receptor, has garnered considerable interest because of its notable and expeditious antidepressant properties observed in individuals diagnosed with major depressive disorder (MDD) who exhibit resistance to conventional therapeutic interventions. A comprehensive and rigorous systematic review was undertaken to evaluate the prevalence of ketamine abuse undergoing ketamine treatment for depressive disorders. A comprehensive search was conducted across the electronic databases to identify pertinent studies published between 2021 and 2023. The present investigation incorporated a comprehensive range of studies encompassing the abuse or misuse of ketamine, including case reports, observational studies, and clinical trials. Data extraction and quality assessment were conducted in accordance with predetermined criteria. The findings of this systematic review demonstrate the importance of monitoring and addressing ketamine abuse in patients receiving ketamine treatment for depressive disorders like MDD. The wide range of reported prevalence rates highlights the need for standardized criteria and measures for defining and assessing ketamine abuse. This study presents a significant contribution to the field by introducing a novel screening questionnaire and assessment algorithm designed to identify and evaluate ketamine misuse among major depressive disorder (MDD) patients undergoing ketamine treatment. This innovative tool holds the potential to enhance clinical practice by providing healthcare professionals with a standardized approach to promptly detect and address ketamine misuse. The integration of this screening tool into routine care protocols can facilitate more effective monitoring and management of ketamine misuse in this population, ultimately leading to improved patient outcomes and safety.

## Introduction

Several studies have shown that ketamine has strong and rapid antidepressant effects in patients with major depressive disorder (MDD) [1, 2]. Ketamine is becoming a popular therapy because it quickly relieves symptoms of depression. However, besides its therapeutic advantages, ketamine misuse is a potential problem in this population [3]. Ketamine misuse refers to the inappropriate or non-medical use of ketamine, where individuals use the substance outside legitimate medical purposes or in doses or frequencies other than prescribed. Misuse can include using ketamine to achieve a high for recreational purposes or in ways not intended by healthcare professionals [4]. Ketamine misuse can lead to various negative consequences, including addiction and cognitive impairment. Healthcare professionals should closely monitor ketamine administration to ensure its appropriate use and minimize the risk of abuse. Ongoing research aims to develop alternative treatments with similar rapid antidepressant effects but without the potential for misuse associated with ketamine.

Ketamine abuse can result in various negative outcomes, particularly when used at higher dosages. Ketamine abuse represents a more severe form of misuse, characterized by recurrent and problematic use despite negative consequences. This includes patterns of ketamine use that lead to significant impairments in daily functioning, psychological dependence, and potential physical harm [5]. Memory deficiencies and concentration issues are among the cognitive impairments linked to persistent ketamine misuse, according to recent research [4]. Ketamine use over an extended period has also been associated with the emergence of psychiatric conditions such as depersonalization-derealization disorder and hallucinogen persistent perception disorder (HPPD) [5]. These results underline the importance of thoroughly monitoring and treating the potential dangers of ketamine use in patients with MDD.

The psychotomimetic properties of ketamine have increased its potential for abuse. Ketamine is appealing for recreational use because it can cause dissociation and psychedelic experiences [6]. However, this also raises the possibility of abuse and complicates the treatment of patients with MDD. The possibility of abuse has been made much more likely by ketamine's accessibility outside medical settings. The illegal use of ketamine has increased, especially among young adults and those looking to treat their depressive symptoms [7]. The potential for misuse in this population is worsened by the simplicity of ketamine administration. Furthermore, ketamine abuse can occur when it diverts from its intended medicinal purposes. Since there is a chance that ketamine will be abused, it is typically only delivered in therapeutic settings under close supervision. There have been cases of people attempting to obtain ketamine from medical sources for purposes other than those for which it was prescribed [8]. Such a diversion jeopardizes patient safety and highlights the necessity for strict ketamine use supervision and regulation.

Healthcare providers must be attentive to monitoring patients' use of ketamine because of the risk of abuse and addiction. To protect patients' well-being, a recent study routinely checked for indicators of abuse and dependence while undergoing ketamine treatment [9]. By implementing these measures, healthcare providers can maintain a comprehensive approach to patient care and help mitigate the potential negative consequences of ketamine use. The implementation thorough screening methods and monitoring systems can make it easier to identify abuse early and enable the necessary actions. In conclusion, ketamine abuse among individuals utilizing ketamine for this purpose cannot be ignored, even though ketamine is a promising drug for successful treatment of MDD. According to recent studies, the risk of abuse is influenced by cognitive deficits, psychotomimetic effects, availability outside medical settings, and diversion from medical use. Healthcare practitioners should prioritize monitoring and regulating ketamine use to reduce the risk of misuse among MDD patients [10, 11].

## Methodology

### Search strategy

This study was conducted following the Preferred Reporting Items for Systematic Reviews and Meta-Analysis (PRISMA) guidelines, which are used extensively to conduct effective research [10]. The literature-based search for this article was conducted on scientific databases such as the National Library of Medicine (Pubmed), Medline-Ovid, Cochrane Central Register of Controlled Trials (CENTRAL) of Cochrane Library along with Pubmed Central (PMC), and Google Scholar with the utilization of the following medical subject heading (MeSH) such as ‘Ketamine Abuse’, ‘Ketamine misuse in bipolar disorder’, ‘ketamine in major depressive disorder’, ‘bipolar depressive disorder’, ‘unipolar depressive disorder’, and ‘treatment-resistant disorder’ along with many more.

### Inclusion criteria

The Studies included in our search focused on ketamine usage among patients with psychiatric disorders, particularly major depressive disorder or bipolar disorder.

Only studies published between 2012 and 2023 were considered, with a preference for those available in English.

### Exclusion criteria

Studies not available in English or lacking full-text accessibility were excluded.

Studies did not include the ketamine use or those published before 2012 were also excluded.

### Eligibility & extraction

**Fig. 1:**
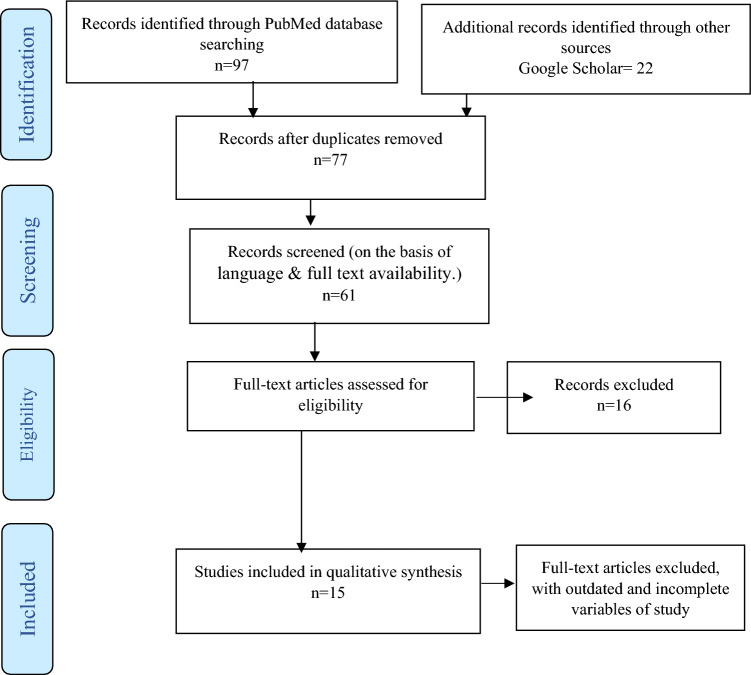
PRISMA diagram for studies selection, eligibility, and data extraction

The titles and abstracts were initially screened to assess study eligibility based on the criteria outlined above. English-language articles were preferred, and randomized controlled trials were preferred. Review articles, case series, and case reports were also included. Eligible articles underwent a thorough full-text review and analysis, according to the Preferred Reporting Items for Systematic Reviews and Meta-Analysis (PRISMA) guidelines (Fig. 1).

### Quality assessment using Newcastle Ottawa Scale (NOS)

The quality of included studies was assessed using the Newcastle Ottawa Scale (NOS), a widely recognized tool for evaluating the methodological quality of non-randomized studies in systematic reviews and meta-analyses [11]. The NOS assesses studies based on three main criteria: selection of study groups, comparability of groups, and ascertainment of the exposure or outcome of interest.

Each study was independently evaluated by two authors to determine its quality rating on the NOS. Discrepancies in ratings were resolved through consensus or consultation with a third author. Studies were categorized into two quality levels based on the NOS score:

Excellent: studies with a high methodological quality and minimal risk of bias across all evaluated criteria.

Fair: studies with some limitations in methodological quality but still providing valuable information.

The NOS findings for each included study are summarized in (Table 1), highlighting key quality indicators such as selection of study participants, comparability of study groups, and outcome assessment.

**Table 1:** Newcastle Ottawa Scale for the eligible articles

	Dean et al. 2021 [12]	Dean RL et al. 2021 [41]	Zarate et al. 2012 [16]	Grunebaum et al. 2017 [42]	Mkrtchain et al. 2021 [43]	Liu et al. 2021 [15]	Hassan et al. 2021 [14]	Findeis et al. 2020 [17]	Farmer et al. 2020 [44]	Chubbs et al. 2022 [40]	Swainson et al. 2022 [45]	Gałuszko et al. 2021 [28]	Ekstrand et al. 2022 [31]	Cosci et al. 2020 [46]	Wtodarczyk et al. 2021 [47]
Representativeness of the exposed cohort	1	1	1	1	1	1	1	1	1	1	1	1	1	1	1
Selection of non-exposed cohort	1	1	1	1	1	1	1	1	1	0	0	1	1	1	1
Ascertainment of exposure	1	1	1	1	1	1	1	1	1	1	1	1	1	1	1
Demonstration that outcome of interest was not present at the start of the study	1	1	1	1	1	1	1	1	1	1	1	1	1	1	1
Comparability of cohorts on analysis or design basis	1	1	1	1	1	1	1	1	1	1	1	1	1	1	1
Outcome assessment	1	1	1	1	1	1	1	1	1	1	1	1	1	1	1
Follow-up duration is enough for an outcome to occur	1	1	1	1	1	1	1	1	1	1	1	1	1	1	1
Adequacy of follow-up cohorts	1	1	1	1	1	1	1	1	1	1	1	1	1	0	1
Total score	8	8	8	8	8	8	8	8	8	8	7	8	8	7	8
Results	Good	Good	Good	Good	Good	Good	Good	Good	Good	Fair	Fair	Good	Good	Fair	Good

## Results

The papers selected for the study were reviewed, and (Table 2) provides the details of data extracted from the research articles. Studies have indicated that ketamine usage is positively correlated with reducing the symptoms of depressive episodes and decreasing suicidal scoring or ideation, primarily MADRS score.

**Table 2:** Characteristics of Research articles included in the study

Study	Year	Location	N	Type of study	Primary diagnosis	Diagnosis criteria	Comorbidity	Follow up time	Outcome
Dean et al. [12]	2021	UK	5299	Review article	UMDD with TR	DSM/RDC/ICD-10	Bipolar depression		27.4% difference observed in symptoms with ketamine at 24 h with the scoring of HDRS, HDRS-1, MADRS
Dean et al. [41]	2021	UK	647	Review article	Bipolar depression/TRBD	DSM/RDC/ICD-10	Unipolar depression		A single dose of ketamine proved to be better than a placebo with an anticipated effect of 10 more per 1000 study population
Zarate et al. [16]	2012	USA	15	Double-blind, randomized, placebo-controlled trial	TRBD	MADRS score	Anxiety disorder/psychosis	4 weeks	Depressive symptoms, as well as suicidal thoughts, were reduced significantly in patients with ketamine as compared to a placebo because 79% of subjects responded to ketamine
Grunebaum et al. [42]	2017	USA	16	Pilot midazolam-controlled randomized clinical trial	MDD with BD	DSM-IV		6 months	ketamine infusion was almost 6 points greater than midazolam in reduction of SSI score in bipolar depressive patients
Mkrtchain et al. [43]	2021	USA	58	A double-blind trial, placebo-controlled, crossover	MDD with TRD	Resting-state Functional magnetic resonance imaging	Leukaemia, dyslipidemia	2 weeks	Ketamine was reported to elevate front striatal connectivity and function in TRD participants, whereas it disrupted that in healthy volunteers
Liu et al. [15]	2021	China	1	Case reports	Autoimmune hepatitis, chronic cholecystitis, cholangitis	CT	Pneumonia, central motor and sensory conduction disorder	Till death	Ketamine misuse/abuse for a prolonged period reported significant brain atrophy, encephalopathy, liver and gall bladder malfunctioning, and urinary dysfunction
Hassan et al. [14]	2022	USA	664	Retrospective study	MDD with TRD	DSM-5 criteria	Anxiety	4 weeks	Ketamine dosage led to decreased scoring of GAD and PHQ-9, depicting ketamine as clinically effective and safe medication for reducing anxiety and depression in non-hospital settings
Findeis et al. [17]	2020	Germany	25	Retrospective Cohort Observational Study	Depressive disorder, BD, schizoaffective disorder	ICD-10 criteria	Peripheral polyneuropathy, PTSD, EIPD, atrial fibrillation,	28 months	The study depicted the absence of urothelial toxicity about repeated intake of eksetamine, the ketamine derivative
Farmer et al. [44]	2020	USA	57	Randomized, placebo-controlled, crossover trial	MDD, bipolar disease with TRD			Six years	The ketamine concentration was positively associated with the prediction of distal antidepressant response along with higher hydroxynorketamine concentration relation in the body with the weaker response of antidepressant medications indicating gamma power changes in humans
Chubbs et al. [40]	2022	Canada	33	Survey based study	TRD				Participants disclosed rescued to no ketamine cravings, whereas just one patient reported a past of unauthorized ketamine consumption, which did not necessarily result in a propensity to misuse prescribed ketamine
Swainson et al. [45]	2022	Canada		Review article	TRD				Ketamine's introduction into psychiatric practice presents challenges due to limited availability and prone to being an addictive substance, but Prompt interventions for treatment-resistant depression and careful administration of ketamine dosage are advised
Gałuszko et al. [28]	2021	Poland	5	Case series	MDD with TRD	MADRS score	HTN, cerebrovascular incident, OCD	35 days	There was efficacy and safety of repeated intake of IV ketamine treatment in TRD in bipolar disorder type I and MDD
Ekstrand et al. [31]	2022	Sweden	186	Randomized, open-label, non-inferiority trial	Unipolar depression	MADRS score		12 months	Remission and overall symptom reduction were observed with multiple ketamine infusions instead of ECT in critically ill individuals
Cosci et al. [46]	2020	Canada		Review article	TRD in psychotic disorders				Ketamine abuse shows symptoms like craving, dysphoria, autonomic arousal, palpitation, mood changes, paranoia, low motor skill agitation, etc. Cognitive impairment may also occur within 24 h of discontinuance with persistence for up to 2 weeks
Wtodarczyk et al. [47]	2021	Poland	49	Exploratory observational study	MDD with TRD or BPD	MADRS score	HTN, MDD, DM, epilepsy hyperlipidemia, myocardial infarct,	1 week	35 were suffering from MDD, which showed 88.9% (n = 8) responders and 62.5% (n = 5) remitters with 68.8% (n = 22) non-responders. 14 bipolar patients, 11% (n = 2) ketamine responders & 37.5% (n = 5) remitters & rest were non-responders. CADSS and BPRS scores indicated no significant differences before or after the analysis for ketamine treatment, which could be due to the small sample size and coexisting treatments

## Discussion

The ketamine derivative of phenylene is used as a rapid-acting intravenous drug along with its sustained antidepressant effect on the human body, mainly in depressive disorders, both major and minor. It is known to positively affect depression and suicidal ideation in patients [12].

### Clinical applications of ketamine

Ketamine has been used in the treatment of several psychiatric conditions, such as major depressive disorder (MDD), treatment-resistant depression (TRD), bipolar disorder (BD), and post-traumatic stress disorder (PTSD). Subanesthetic dosages of ketamine elicit prompt antidepressant and antisuicidal effects in individuals diagnosed with Major Depressive Disorder (MDD), Treatment-Resistant Depression (TRD), and Bipolar Disorder (BD). The FDA granted approval for the use of esketamine nasal spray (Spravato^®^) for the treatment of treatment-resistant depression (TRD). Although ketamine shows potential as a treatment for various diseases, its psychoactive side effects have hindered its widespread use. This finding highlights the need to thoroughly understand the potential for addiction [13].

However, its misuse can lead to non-specific clinical symptoms ranging in the extent of symptom appearance, which can be mild or chronic. Some signs include biliary dilation, abnormality in liver function, hydronephrosis, and cholangitis in some cases. It was suggested that after a thorough investigation of ketamine usage during depressive disorders, it might result in an elevated response and remission ratio with a reduction in rating scale scores over placebo or midazolam [12].

### Therapeutic effects of ketamine in depressive disorders

Antidepressants and anti-suicidal drugs are prescribed to them owing to the fact that a significant portion of their life is spent in depression and pessimistic thoughts. Major depressive disorder, specifically treatment-resistant depression, whose etiology is not known, was reviewed for the purpose of this study. The ventral prefrontal cortex (PFC) and striatum, the primary reward circuits of the brain were observed, and proposed that ketamine elevates the functional connectivity of the frontal striatal circuit of TRD.

The ventral prefrontal cortex (PFC) and striatum, the primary reward circuits of the brain were observed, and proposed that ketamine elevates the functional connectivity of the frontal striatal circuit of TRD. Ketamine usage was correlated with sustained improvement in patients with TRD, along with stability of frontal striatal connections in their brains.

### Clinical presentation, diagnosis, and management of Major Depressive Disorder (MDD)

The study group belonged to the age category of adults, mainly 18 years or older. The studies by Dean et al. [12] and Hassan et al. [14] consisted of larger sample sizes of 5299 and 664, respectively. Larger cohorts indicate reliable results and validity of inferences. The studies included were predominantly control trials with placebo or other factors controlled to ensure the gold standard of research utilized to derive this outcome.

Major depressive disorders are reported to be the ones with a lack of energy and persistently pessimistic behavior, along with complaints of anhedonia, agitation, and sleep issues [12, 15]. Suicidal thoughts are also holding prominence in major depressive disorder [16]. Antidepressants, along with other psychological and pharmacological therapeutic methodologies, are used to overcome depression and remission among patients with MDD. The constant use of medications causes resistance to the treatment of depression. In contrast, first line of antidepressants, such as NMDAR antagonists, are not always beneficial to the patient. If two or more antidepressant medications do not provide aid to patients with depression, their depression is marked as treatment-resistant depression with electroconvulsive therapy or other severe measures to be used for ease of symptoms. Such treatments are available to make patients with MDD feel better, along with the urge to increase their quality of life by the team of professionals adept at handling such cases [17].

Bipolar disorder, along with MDD, is among the classes of psychiatric diseases requiring constant care and management, as a significant portion of a patient's life is spent in depression and the urge to fight it. Treatment resistance and failure are significantly higher in patients with bipolar disorder than in those with other types of MDD [12]. We utilized patients with bipolar disorder in many studies alongside other psychiatric disorders, such as unipolar disorder. The diagnostic criteria used vary among patients within MDD. Some studies utilized the DSM-IV, DSM-V, ICD-10, and many different scales to diagnose bipolar, unipolar, or major depressive disorders among individuals [17].

### Ketamine use in MDD

Ketamine has garnered significant attention for its remarkable efficacy in treating MDD, especially treatment-resistant depression (TRD). Numerous clinical trials and meta-analyses have demonstrated the rapid onset of antidepressant effects, often within hours to days of administration. For instance, a meta-analysis pooled data from several randomized controlled trials (RCTs) and reported a significant reduction in depressive symptoms within 24 h of ketamine infusion [18]. Zarate et al. [19] showed sustained antidepressant effects lasting up to several weeks post-treatment [19].

While ketamine's efficacy is promising, its use in MDD necessitates careful consideration of safety and tolerability. Short-term adverse effects commonly include dissociation, transient increase in blood pressure, and cognitive impairment. Long-term concerns such as the potential for abuse and cognitive decline also warrant attention. To mitigate risks, clinicians implement dose optimization strategies and closely monitor patients during and after ketamine administration [20].

Patient selection criteria for ketamine therapy typically include a diagnosis of MDD, failure to respond to conventional antidepressant treatments, and absence of contraindications such as psychosis or unstable medical conditions. Dosing regimens vary, but often involve intravenous infusion of ketamine at subanesthetic doses, with treatment frequency ranging from weekly to monthly. However, the emergence of alternative administration routes, such as intranasal ketamine, offers flexibility in treatment delivery and may improve patient adherence. Daly et al. [21] compared intravenous and intranasal ketamine and found comparable efficacy and tolerability between the two routes [21].

Ongoing research seeks to optimize ketamine's therapeutic potential in MDD and address unresolved issues. Novel formulations, including esketamine nasal spray, represent an advancement in treatment delivery and have received regulatory approval for TRD. Combination therapies involving ketamine and traditional antidepressants or psychotherapy are being investigated to enhance treatment outcomes and prolong remission. However, challenges persist, such as determining optimal maintenance strategies to sustain antidepressant effects and identifying reliable predictors of treatment response. Collaborative efforts between clinicians, researchers, and pharmaceutical companies are crucial for advancing ketamine-based therapies and improving outcomes for individuals with MDD.

### Ketamine effect on the brain

Ketamine operates primarily at the molecular level as an antagonist of the N-methyl-D-aspartate (NMDA) receptor. This interaction disrupts glutamatergic neurotransmission, leading to downstream effects on neural signaling and circuitry in the brain. By blocking NMDA receptors, ketamine alters the balance between excitatory and inhibitory neurotransmission, which is crucial for various cognitive and emotional processes [22]. One of the most intriguing aspects of ketamine's mechanism is its potential to enhance neuroplasticity and promote synaptic connectivity. This phenomenon is particularly relevant for the observed antidepressant effects. Studies have suggested that ketamine administration can stimulate the growth of new synaptic connections and facilitate the remodeling of existing neural circuits. These changes may underlie the rapid and sustained improvements in mood observed after ketamine treatment.

Research has highlighted the effect of ketamine on key brain regions involved in mood regulation, including the prefrontal cortex, hippocampus, and amygdala. A study by Reed et al. [23] used functional magnetic resonance imaging (fMRI) to demonstrate that ketamine administration led to increased activation in the prefrontal cortex, a region associated with emotional regulation and cognitive control [23]. Another study by Rawat et al. [24] utilized structural MRI to show that ketamine induced rapid and transient increases in hippocampal volume, suggesting a potential role in promoting neurogenesis and synaptic plasticity [24]. Scheidegger et al. [25] found that ketamine administration reduced amygdala hyperactivity, a common feature of mood disorders, and restored normal fear responses [25].

In addition to its actions on NMDA receptors, ketamine modulates various neurotransmitter systems, including glutamate, gamma-aminobutyric acid (GABA), and monoamines such as serotonin and dopamine. Ketamine's effects on these neurotransmitters play a crucial role in shaping its therapeutic profile and may contribute to its efficacy in treating mood disorders. A study by Aleksandrova et al. [26] demonstrated that ketamine's rapid antidepressant effects were dependent on its ability to increase glutamate release and activate α-amino-3-hydroxy-5-methyl-4-isoxazolepropionic acid (AMPA) receptors [26].

Ketamine exerts its effects on the brain through a multifaceted mechanism involving NMDA receptor antagonism, modulation of neurotransmitter systems, and promotion of neuroplasticity. Understanding these neurobiological mechanisms is essential for elucidating ketamine's therapeutic potential and developing novel treatments for mood disorders.

### Assessment of ketamine treatment efficacy, and clinical outcomes

The studies utilized MADRS scoring primarily to determine the effect of ketamine by assessing and recording the scores before and after treatment for optimal comparison and analysis. One study showed that a few dosages, such as three intakes of ketamine therapy, reported a positive improvement in the severity of depressive episodes along with an improvement in PHQ-9 and GAD-7 scores in half of the patients. Scores improved by up to 60% in patients who completed the six ketamine therapy courses recommended clinically. The anxiety category with moderate to severe category patients showed a reduction in anxiety after three dosages with GAD-7 score improvement and a 50% reduction in scores compared to the scores at baseline of the study [14].

Bryant et al. [27] characterized a group of six elderly patients who had received short and long-term maintenance phases of ketamine infusions. Five of six patients demonstrated a significant reaction, indicating the promising effects of ketamine infusion [28].

### Analogues of ketamine

Analogues Analogs of ketamine represent a diverse array of compounds that share structural similarities or pharmacological properties with ketamine etsketamine (S)-ketamine and R enantiomer (R)-ketamine (arketamine), two notable analogs of ketamine. Esketamine is the S-enantiomer of ketamine, a dissociative anesthetic agent originally developed in the 1960s. It is a non-competitive NMDA receptor antagonist that blocks the action of N-methyl-D-aspartate (NMDA) receptors in the brain. Arketamine is the R-enantiomer of ketamine. Similar to esketamine, it is a non-competitive NMDA receptor antagonist. Although esketamine has been developed and approved for the treatment of depression, its potential therapeutic effects are still being investigated [29].

Esketamine has garnered attention for its rapid and robust antidepressant effects, leading to FDA approval for the treatment of Major Depressive Disorder (MDD). Conversely, arketamine, an enantiomer of ketamine, has also demonstrated promising effects in MDD treatment. In an open-label pilot trial, seven participants with Treatment-Resistant Depression (TRD) who received only one intravenous infusion provided data on arketamine. Administration of 0.5 mg/kg arketamine resulted in a decrease in the Montgomery-Asberg Depression Rating Scale (MADRS) scores with minimal signs of dissociation. This distinction between esketamine (S)-ketamine and arketamine is essential, as both enantiomers may offer unique therapeutic benefits and safety profiles in the treatment of MDD [28]. Wei et al. asserted that NMDAR is frequently involved in the mechanism by which antidepressants function with arketamine. However, the principal biochemical mechanism by which ketamine functions to treat depression is not known [30]. 

### Ketamine in electroconvulsive therapy

Electroconvulsive therapy (ECT), the most effective treatment for depressive disorders, and ketamine were compared. There is an abundance of interest in the findings of a glutamate-modulating pharmaceutical that reduced depression-related symptoms and enabled patients to recuperate within hours of receiving a single IV Subanesthetic dosage, so they compared it to electroconvulsive therapy to investigate the reliability of ketamine effect in MDD or TRD. Among patients receiving ECT, 63% of patients remitted and felt better, whereas 45% of ketamine-treated patients remitted, indicating that the remission rate was higher in the ECT group with 47/94(61%) patients remitted, and 44/97 (45%) remitted in ketamine-treated group (chi^2^ = 4.5, P = 0.034, 95% CI [1.1%, 29]) [31].

### Ketamine in pediatric usage

Ketamine is widely used as a pediatric anesthetic drug during medical procedures. Repeated exposure to subanesthetic doses of ketamine can result in extensive neuronal degeneration in the developing nervous system of neonates [32]. However, it is unknown whether this might cause cognitive impairment in the adult brain. NMDA receptors in the central nervous system play a role in myelination, which is essential for the development of neurons and optimal cognitive functioning [33]. However, it is unclear whether ketamine exposure during brain development can inhibit myelination and subsequently impair cognitive function in adults. This issue should be researched to address concerns regarding the use of ketamine in pediatric anesthesia.

The findings of this study demonstrated that ketamine, prenatal exposure to the experimental setting, or both enhanced MBP transcription in the mPFC of adult female rats. Ketamine was also shown to cause more MBP expression, which renders it a safe medication for pediatric anesthesia [34].

### Misuse of ketamine

The efficacy of ketamine as an antidepressant medication has been questioned, while adverse outcomes of ketamine treatment in depressed individuals, particularly long-term consequences, remain only partially understood. Acute and temporary side effects are frequent, and include alterations in cognition, hypertension, anxiety, dissociation, and vertigo. Additionally, there are cases of hepatocellular and urinary intoxication among recreational drug users and patients receiving recurrent high dosages of medications for pain [31].

### Ketamine misuse mechanism

Ketamine, a rapid-acting antidepressant, is sustained for up to 7 days in patients with TRD and suicidal patients. Its use was done sparingly for managing TRD and MDD or both, but due to abuse, addiction, and side effects, it has been minimized [15]. The formation of subcortical areas, such as the nucleus accumbens, which is linked to addiction behaviors, is influenced by the disruption of glutamate neurotransmission, which is connected to ketamine addiction. Additionally, studies have demonstrated that NMDA receptor-targeting glutamatergic drugs have distinct antidepressant qualities that effectively reduce suicidal thoughts [35]. γ-Aminobutyric acid (GABA), serotonin, dopamine, opioids, sigma, and cholinergic receptors are some of the additional low-affinity pharmaceutical targets of ketamine, in addition to voltage-gated sodium levels and hyperpolarization-activated cyclic nucleotide-gated channels [36]. This may result in various outcomes resulting from acute to long-term ketamine usage. For instance, GABA is the primary inhibiting neurotransmitter in the human brain, and optimal communication between neurons and cognitive performance depends on a healthy equilibrium between inhibitory and excitatory synaptic transmissions. Convulsions in the brain were the patient's primary symptoms, potentially due to insufficient inhibitory effects such as GABA. However, the functional significance of the impact of ketamine on GABA receptors is unknown [37]. According to a recent study [38], the effect of GluN2B-NMDARs on GABA neurons causes a single sub-anesthetic dose of ketamine to have an immediate and long-lasting antidepressant effect. This suggests that ketamine may not act directly on other receptors (aside from NMDA receptors) but may also have secondary effects. The medication dosage used for abuse is the same as or even higher than the amount used for therapy, which is usually 1–2 mg/kg body weight. Furthermore, extended-period ketamine users might exhibit a decrease in frontal gray matter volume on MRI [39]. Consequently, it was anticipated that researchers would identify cerebral atrophy on CT scans in patients with long-term ketamine abuse. AMPA (α-amino-3-hydroxy-5-methyl-4-isoxazole-propionic acid) receptors are linked to ketamine damage. Additionally, a study found that atypical protein tau phosphorylation at specific locations mediates alterations in membrane AMPA receptors and synaptic functioning caused by ketamine. There is a correlation between addictive substances and alterations in brain volume as reported by Liu et al. [15].

### Consideration of risks and side effects

While ketamine demonstrates significant therapeutic potential in the treatment of depressive disorders, it is not without risks, particularly when used inappropriately or excessively. Understanding and acknowledging these potential adverse effects is crucial for informed decision making in clinical practice.

Prolonged or excessive use of ketamine has been associated with a range of adverse effects on hepatic, urinary, and neurological functioning. Studies have documented hepatic dysfunction, urinary tract abnormalities, and neurological impairments among individuals misusing ketamine [15].

Long-term misuse of ketamine has raised concerns regarding its potential to induce brain atrophy and neurological damage. Chronic ketamine abuse has been linked to structural alterations in the brain, including reductions in gray matter volume and disruptions in neural connectivity [17]. Studies have reported significant brain atrophy and neurological dysfunction in individuals with a history of prolonged ketamine misuse, highlighting the need for caution and vigilance in its use [40].

### Safe intake

Ketamine usage using the prescribed method and design is advised to avoid the adverse effects of ketamine usage. The potential benefits of ketamine in TRD are of significant importance, which can pave the way for the substantial use of ketamine in major depressive disorders, such as unipolar and bipolar depression.

The absence of sufficient data about the addictive potential of ketamine as a psychiatric treatment does not deter its usage when deemed necessary. However, prudence is required, akin to benzodiazepines and stimulants. Medical practitioners should exercise caution when prescribing ketamine while remaining attentive to potential emerging data that could alter the balance between risks and benefits.

### Limitations

The lack of extensive data with the availability of cohort data on this subject limits the study with the recommendation of further observational and cohort studies along with randomized control trials. The combined effect of other drugs with ketamine should also be observed, and enantiomers with decreased risk of addiction or brain atrophy should be developed to increase the quality of life of patients with major depressive disorder who spend a significant amount of time fighting the internal battle of mental health, which is of prime importance.

Our systematic review acknowledges several potential biases that could affect the interpretation of our findings. First, publication bias may have affected our results, as studies with statistically significant or positive outcomes were more likely to be published, potentially skewing our synthesis of evidence. Selection bias could have affected the generalizability of our findings. Although we aimed to include a wide range of studies, certain populations or types of studies may have been disproportionately included in our review. We conducted a comprehensive search using predefined criteria to minimize bias in study selection. The quality and characteristics of the included studies could influence the overall conclusions of our systematic review.

While our review provides valuable insights into ketamine misuse in major depressive disorder patients with MDD, readers should interpret the findings within the context of these acknowledged biases. Future research should address these biases through more rigorous study designs and transparent reporting practices.

### Future implications

**Fig. 2:**
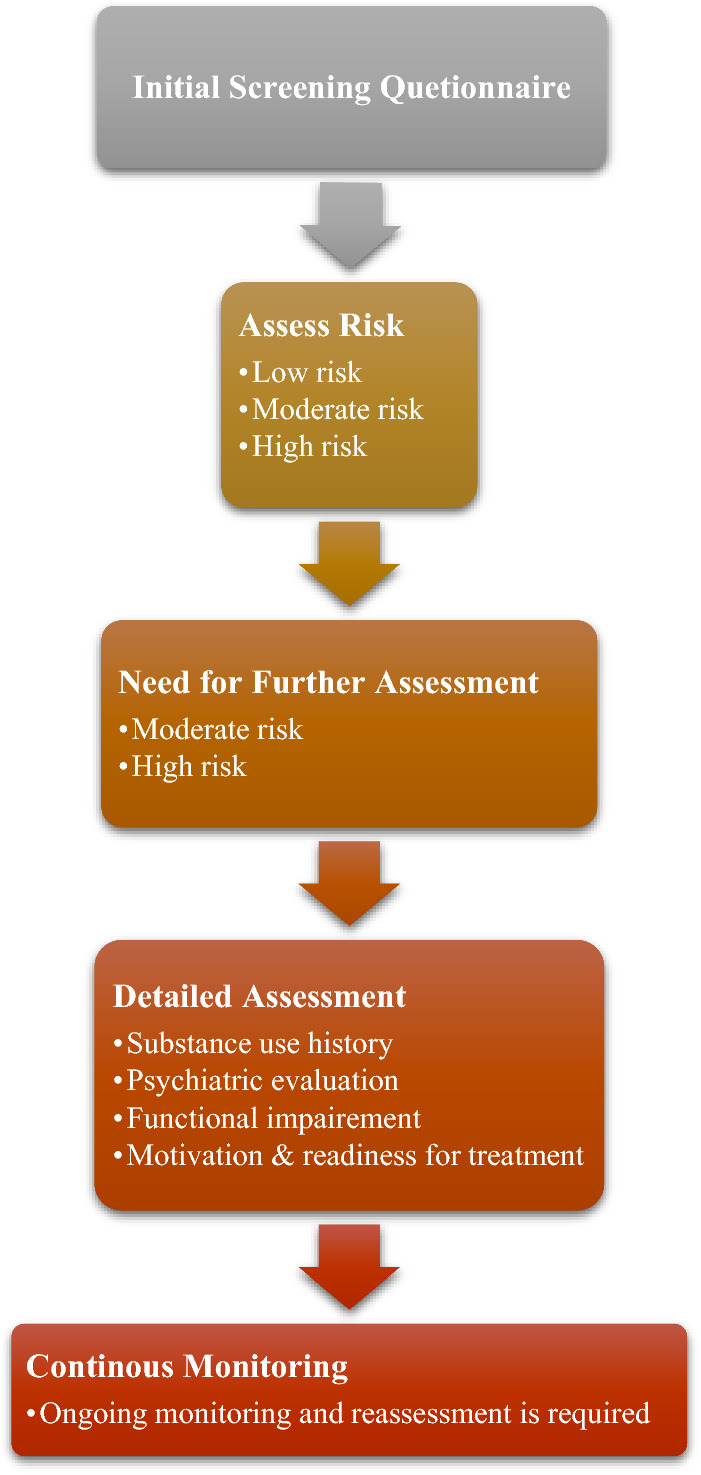
Assessment of patients for ketamine abuse

The correlation between the ketamine abuse and depressive disorders is a multifaceted problem that necessitates inventive approaches to enhance timely identification, tailored treatment strategies, and overall psychological welfare. Progress in technology, including artificial intelligence and machine learning, has the potential to transform the screening procedures for depressive illnesses. Predictive algorithms can examine behavioral patterns, social media activity, and biomarkers to detect the initial indications of depression. The integration of wearable gadgets and smartphone applications has the potential to provide real-time data, leading to more precise treatment. Pharmacological precision medicine can provide guidance for the proper use of ketamine in a closely supervised setting, thereby reducing the likelihood of its misuse. Telemedicine and remote monitoring have the potential to improve the availability of mental health services, making it easier to identify individuals who are at risk of misusing ketamine or experiencing symptoms of depression at an early stage. Integrated care models have the potential to facilitate a more cohesive approach to mental health care by dismantling barriers between primary care, mental health services, and drug misuse. Ethical considerations are vital, as it is essential to strike a balance between technological development and individual privacy. Prudent supervision and guidelines are necessary for ethical administration of ketamine in the management of depressive disorders. By adopting these advancements, we come closer to a more thorough and efficient strategy for dealing with the intricate relationship between ketamine abuse, depressive conditions, and overall psychological welfare. The tools available for the diagnosis of such abuse can also be utilized, as shown in Table 3 and Fig. 2.

**Table 3:** Risk assessment questionnaire

No	Questions	Options
1	Have you ever used ketamine or any ketamine-containing products?	Yes: + 2 No: 0
2	How frequently have you used ketamine in the past?	Occasionally: + 1 Regularly: + 2 Rarely: + 1 Never used: 0
3	Have you ever experienced problems related to ketamine use, such as tolerance, withdrawal symptoms, or unsuccessful attempts to cut down or control use?	Yes: + 2 No: 0
4	Have you ever been diagnosed with substance use disorder (e.g., alcohol, drugs)?	Yes: + 2 No: 0
5	Have you ever been diagnosed with any psychiatric disorders?	Depression: + 1 Anxiety: + 1 Bipolar disorder: + 2 PTSD (Post-Traumatic Stress Disorder): + 1 Schizophrenia: + 2 Other (please specify): + 1 each None: 0
6	Do you have a family history of substance use disorders?	Yes: + 1 No: 0
7	Are you currently receiving treatment for any psychiatric disorders?	Yes: + 1 No: 0
8	Do you have access to ketamine or know someone who does?	Yes: + 1 No: 0
9	Are you seeking ketamine treatment for legitimate medical reasons, such as depression or chronic pain?	Yes: 0 No: + 1
10	On a scale of 1 to 10, how motivated are you to follow prescribed treatment plans and avoid misuse of ketamine?	1 to 3: + 1 4 to 7: 0 8 to 10: −1
	Scoring: 0–5 points: Low risk 6–10 points: Moderate risk 11 or more points: High risk	
	Interpretation: Low risk: minimal to no risk factors identified, may proceed with caution Moderate risk: some risk factors present, may require closer monitoring and intervention High risk: significant risk factors identified, may necessitate alternative treatment options or specialized care

## Conclusion

Ketamine has demonstrated potent and expeditious antidepressant properties in individuals diagnosed with depressive disorders such as Major Depressive Disorder (MDD). However, overuse of ketamine can lead to undesirable outcomes such as addiction and cognitive damage. Healthcare practitioners should diligently oversee the administration of ketamine to guarantee its proper utilization and mitigate the potential for misuse. The abuse of ketamine can lead to a range of adverse consequences, such as cognitive impairment, mental disorders, and physiological ailments. Prolonging and excessive use of ketamine can cause adverse hepatic and urinary effects along with the risk of brain atrophy. The implementation of comprehensive screening procedures and monitoring systems is crucial for promptly detecting instances of abuse and taking appropriate measures. Notwithstanding its potential advantages, ketamine abuse must not be disregarded, and stringent oversight and control are important. This study introduces a valuable contribution to the field through a novel screening questionnaire and assessment algorithm tailored for individuals with MDD undergoing ketamine treatment, offering healthcare professionals a standardized tool to promptly detect and address ketamine misuse. Integrating this innovative approach into routine care protocols enhances monitoring, mitigates risks, and underscores the importance of stringent oversight and control in addressing the complex issue of ketamine abuse in this population.

## Data Availability

No datasets were generated or analysed during the current study.

## Abbreviations

MDD: Major depressive disorder
HPPD: Hallucinogen persistent perception disorder
PRISMA: Preferred reporting items for systematic reviews and meta-analysis
NOS: Newcastle Ottawa scale
UMDD: Unipolar major depressive disorder
RDC: Research diagnostic criteria
DSM: Diagnostic and statistical manual of mental disorders
ICD: International classification of diseases
HDRS: Hamilton depression rating scale
MADRS: Montgomery–Åsberg depression rating scale
TRBD: Treatment-resistant bipolar disease
BD: Bipolar disease
PTSD: Post traumatic stress disorder
TRD: Treatment-resistant depression
HTN: Hypertension
EIPD: Emotionally instable personality disorder
PFC: Pre-frontal cortex
NMDAR: N-methyl D-aspartate receptor
PHQ-9: Patient Health Questionnaire-9
AMPAR: α- Amino-3 hydroxy- 5-methyl -4 isoxazolepropionic acid

## References

[1] Abdallah CG, Averill LA, Krystal JH (2020). Ketamine as a promising prototype for a new generation of rapid-acting antidepressants. Ann N Y Acad Sci.

[2] Singh JB, Fedgchin M, Daly EJ (2016). A double-blind, randomized, placebo-controlled, dose-frequency study of intravenous ketamine in patients with treatment-resistant depression. Am J Psychiatry.

[3] Kishimoto T, Chawla JM, Hagi K (2020). Single-dose infusion ketamine and non-ketamine N-methyl-D-aspartate receptor antagonists for unipolar and bipolar depression: a meta-analysis of efficacy, safety and time trajectories. Psychol Med.

[4] Harborne GC, Watson FL, Healy DT, Groves L (1996). The effects of sub-anaesthetic doses of ketamine on memory, cognitive performance and subjective experience in healthy volunteers. J Psychopharmacol.

[5] Colizzi M, Ruggeri M, Bhattacharyya S (2020). Unraveling the intoxicating and therapeutic effects of cannabis ingredients on psychosis and cognition. Front Psychol.

[6] Muscat SA, Hartelius G, Crouch CR, Morin KW (2021). An Integrative approach to ketamine therapy may enhance multiple dimensions of efficacy: improving therapeutic outcomes with treatment resistant depression. Front Psychiatry.

[7] Yavi M, Lee H, Henter ID, Park LT, Zarate CA (2022). Ketamine treatment for depression: a review. Discov Ment Health.

[8] Siegel AN, Di Vincenzo JD, Brietzke E, Gill H, Rodrigues NB, Lui LMW, Teopiz KM, Ng J, Ho R, McIntyre RS, Rosenblat JD (2021). Antisuicidal and antidepressant effects of ketamine and esketamine in patients with baseline suicidality: a systematic review. J Psychiatr Res.

[9] McIntyre RS, Rosenblat JD, Nemeroff CB (2021). Synthesizing the evidence for ketamine and Esketamine in treatment-resistant depression: an international expert opinion on the available evidence and implementation. Am J Psychiatry.

[10] Page MJ, McKenzie JE, Bossuyt PM, Boutron I, Hoffmann TC, Mulrow CD, Shamseer L, Tetzlaff JM, Akl EA, Brennan SE, Chou R, Glanville J, Grimshaw JM, Hróbjartsson A, Lalu MM, Li T, Loder EW, Mayo-Wilson E, McDonald S, McGuinness LA, Stewart LA, Thomas J, Tricco AC, Welch VA, Whiting P, Moher D (2021). The PRISMA 2020 statement: an updated guideline for reporting systematic reviews. BMJ.

[11] Wells G, Shea B, O’Connell D, Peterson J, Welch V, Losos M, Tugwell P. The Newcastle-Ottawa Scale (NOS) for assessing the quality of nonrandomised studies in meta-analyses. 2013. http://www.evidencebasedpublichealth.de/download/Newcastle_Ottowa_Scale_Pope_Bruce.pdf. Accessed July 2023.

[12] Dean RL, Hurducas C, Hawton K, Spyridi S, Cowen PJ, Hollingsworth S, Cipriani A (2021). Ketamine and other glutamate receptor modulators for depression in adults with unipolar major depressive disorder. Cochrane Database Syst Rev.

[13] Corriger A, Pickering G (2019). Ketamine and depression: a narrative review. Drug Des Dev Ther.

[14] Hassan K, Struthers WM, Sankarabhotla A, Davis P (2022). Safety, effectiveness and tolerability of sublingual ketamine in depression and anxiety: a retrospective study of off-label, at-home use. Front Psychiatry.

[15] Liu L, Huang H, Li Y, Zhang R, Wei Y, Wu W (2021). Severe encephalatrophy and related disorders from long-term ketamine abuse: a case report and literature review. Front Psychiatry.

[16] Zarate CA, Brutsche NE, Ibrahim L, Franco-Chaves J, Diazgranados N, Cravchik A (2012). Replication of ketamine's antidepressant eBicacy in bipolar depression: a randomized controlled add-on trial. Biol Psychiat.

[17] Findeis H, Sauer C, Cleare A, Bauer M, Ritter P (2020). Urothelial toxicity of ketamine in the treatment of depression. Psychopharmacology.

[18] Caddy C, Amit BH, McCloud TL, Rendell JM, Furukawa TA, McShane R (2015). Ketamine and other glutamate receptor modulators for depression in adults. Cochrane Database Syst Rev.

[19] Zarate CA, Singh JB, Carlson PJ, Brutsche NE, Ameli R, Luckenbaugh DA, Manji HK (2006). A randomized trial of an N-methyl-D-aspartate antagonist in treatment-resistant major depression. Arch Gen Psychiatry.

[20] Phillips JL, Norris S, Talbot J, Birmingham M, Hatchard T, Ortiz A, Blier P (2019). Single, repeated, and maintenance ketamine infusions for treatment-resistant depression: a randomized controlled trial. Am J Psychiatry.

[21] Daly EJ, Singh JB, Fedgchin M, Cooper K, Lim P, Shelton RC, Manji H (2018). Efficacy and safety of intranasal esketamine adjunctive to oral antidepressant therapy in treatment-resistant depression: a randomized clinical trial. JAMA Psychiat.

[22] Xu S, Yao X, Li B, Cui R, Zhu C, Wang Y, Yang W (2022). Uncovering the underlying mechanisms of ketamine as a novel antidepressant. Front Pharmacol.

[23] Reed JL, Nugent AC, Furey ML, Szczepanik JE, Evans JW, Zarate CA (2019). Effects of ketamine on brain activity during emotional processing: differential findings in depressed versus healthy control participants. Biol Psychiatry Cogn Neurosci Neuroimaging.

[24] Rawat R, Tunc-Ozcan E, Dunlop S, Tsai Y-H, Li F, Bertossi R, Peng C-Y, Kessler JA (2024). Ketamine’s rapid and sustained antidepressant effects are driven by distinct mechanisms. Cell Mol Life Sci.

[25] Scheidegger M, Henning A, Walter M, Lehmann M, Kraehenmann R, Boeker H, Seifritz E, Grimm S (2016). Ketamine administration reduces amygdalo-hippocampal reactivity to emotional stimulation. Hum Brain Mapp.

[26] Aleksandrova LR, Phillips AG, Wang YT (2017). Antidepressant effects of ketamine and the roles of AMPA glutamate receptors and other mechanisms beyond NMDA receptor antagonism. J Psychiatry Neurosci.

[27] Le TT, Cordero IP, Jawad MY, Swainson J, Di Vincenzo JD, Jaberi S, Phan L (2022). The abuse liability of ketamine: a scoping review of preclinical and clinical studies. J Psychiatr Res.

[28] Gałuszko-Wȩgielnik M, Włodarczyk A, Cubała WJ, Wilkowska A, Górska N, Słupski J (2021). Case report: repeated series of ketamine infusions in patients with treatment-resistant depression: presentation of five cases. Front Psychiatry.

[29] Jelen LA, Young AH, Stone JM (2021). Ketamine: a tale of two enantiomers. J Psychopharmacol.

[30] Bryant KA, Altinay M, Finnegan N, Cromer K, Dale RM (2019). Effects of repeated intravenous ketamine in treatment-resistant geriatric depression. A case series. J Clin Psychopharmacol.

[31] Ekstrand J, Fattah C, Persson M, Cheng T, Nordanskog P, Åkeson J, Tingström A, Lindström MB, Nordenskjöld A, Movahed RP (2022). Racemic ketamine as an alternative to electroconvulsive therapy for unipolar depression: a randomized, open-label, non-inferiority trial (KetECT). Int J Neuropsychopharmacol.

[32] Wei Y, Chang L, Hashimoto K (2021). Molecular mechanisms underlying the antidepressant actions of arketamine: beyond the NMDA receptor. Mol Psychiatry.

[33] Dong C, Anand KJS (2013). Developmental neurotoxicity of ketamine in pediatric clinical use. Toxicol Lett.

[34] Zhang J, Chen B, Deng X, Wang B, Liu H (2019). Neonatal exposure to the experimental environment or ketamine can induce long-term learning dysfunction or overmyelination in female but not male rats. NeuroReport.

[35] Lundgaard I, Luzhynskaya A, Stockley JH, Wang Z, Evans KA, Swire M (2013). Neuregulin and BDNF induce a switch to NMDA receptor-dependent myelination by oligodendrocytes. PLoS Biol.

[36] Zhou YL, Wu FC, Liu WJ, Zheng W, Wang CY, Zhan YN (2020). Volumetric changes in subcortical structures following repeated ketamine treatment in patients with major depressive disorder: a longitudinal analysis. Transl Psychiatry.

[37] Zanos P, Gould TD (2018). Mechanisms of ketamine action as an antidepressant. Mol Psychiatry.

[38] Zanos P, Moaddel R, Morris PJ, Riggs LM, Highland JN, Georgiou P (2018). Ketamine and ketamine metabolite pharmacology: insights into therapeutic mechanisms. Pharmacol Rev.

[39] Gerhard DM, Pothula S, Liu RJ, Wu M, Li XY, Girgenti MJ (2020). GABA interneurons are the cellular trigger for ketamine’s rapid antidepressant actions. J Clin Invest.

[40] Chubbs B, Wang J, Archer S, Chrenek C, Khullar A, Wolowyk M, Swainson J (2022). A survey of drug liking and cravings in patients using sublingual or intranasal ketamine for treatment resistant depression: a preliminary evaluation of real world addictive potential. Front Psychiatry.

[41] Dean RL, Marquardt T, Hurducas C, Spyridi S, Barnes A, Smith R, Cipriani A (2021). Ketamine and other glutamate receptor modulators for depression in adults with bipolar disorder. Cochrane Database of Syst Rev.

[42] Grunebaum MF, Ellis SP, Keilp JG, Moitra VK, Cooper TB, Marver JE (2017). Ketamine versus midazolam in bipolar depression with suicidal thoughts: a pilot midazolamcontrolled randomized clinical trial. Bipolar Disord.

[43] Mkrtchian A, Evans JW, Kraus C, Yuan P, Kadriu B, Nugent AC, Roiser JP, Zarate CA (2021). Ketamine modulates fronto-striatal circuitry in depressed and healthy individuals. Mol Psychiatry.

[44] Farmer CA, Gilbert JR, Moaddel R, George J, Adeojo L, Lovett J, Nugent AC, Kadriu B, Yuan P, Gould TD, Park LT, Zarate CA (2020). Ketamine metabolites, clinical response, and gamma power in a randomized, placebo-controlled, crossover trial for treatment-resistant major depression. Neuropsychopharmacology.

[45] Swainson J, Klassen LJ, Brennan S, Chokka P, Katzman MA, Tanguay RL, Khullar A (2022). Non-parenteral ketamine for depression: a practical discussion on addiction potential and recommendations for judicious prescribing. CNS Drugs.

[46] Cosci F, Chouinard G (2020). Acute and persistent withdrawal syndromes following discontinuation of psychotropic medications. Psychother Psychosom.

[47] Wtodarczyk A, Cubata W, Galuszko-Wegielnik M, Szarmach J (2021). Dissociative symptoms with intravenous ketamine in treatment-resistant depression exploratory observational study. Medicine.

